# A Holistic Ecological Approach to Injury Prevention and Management in Youth Sport: A Scoping Review

**DOI:** 10.1111/sms.70341

**Published:** 2026-07-17

**Authors:** Nicklas Balle Venzel, Kristoffer Henriksen, Merete Møller, Louise Kamuk Storm

**Affiliations:** ^1^ Department of Sports Science and Clinical Biomechanics University of Southern Denmark Odense Denmark; ^2^ Oslo Sports Trauma Research Center, Department of Sports Medicine Norwegian School of Sports Sciences Oslo Norway

**Keywords:** environment, interpersonal, psychosocial, rehabilitation, sport injuries

## Abstract

Injuries are a major concern in youth sport. Traditionally, injury research has focused on biomedical and psychological characteristics of the individual athlete. However, newer trends suggest that injuries result from dynamic interaction between the athletes and their surroundings. The objective of this scoping review was to identify positive and negative environmental factors associated with injury prevention and management in youth sport. This scoping review was conducted according to guidelines of The Joanna Briggs Institute. We systematically searched five databases in November 2024 and updated in October 2025. A total of 39 studies were selected of which five were reviews. Of the remaining studies, 23 used qualitative, five used quantitative, and six used mixed‐method designs. Based on the Holistic Ecological Approach, 40 factors were identified and categorized into preconditions (e.g., lack of resources and equipment), processes (e.g., support from peers), structure (e.g., busy training/match schedule), and culture (e.g., culture of risk‐taking). In all four categories, both positive and negative factors were identified, although negative influences predominated. Our findings support the importance of environmental factors in youth sport injury prevention and management, yet these factors have so far been studied in isolation rather than as parts of the broader environment. Collectively, they provide an initial sense of how the environment facilitates injury prevention and management, but based on the current literature, we cannot say how these factors work together. Future research should examine how the identified factors interact and cohesively influence injuries in youth sport.

Youth sport participation can be associated with significant health benefits [1, 2], but does also pose a serious concern as the leading cause of musculoskeletal injuries [3, 4, 5]. A previous study in youth handball players 14–18 years of age indicated that almost half of the players in the youngest age group had experienced a previous injury 1 year prior to study start [6]. A Swedish study showed that over half of 284 youth sport athletes from different sports reported at least one new injury in the past year, and that, on average, about 30% reported an injury every week during a 1‐year period [7]. Several injury prevention programs have been shown to reduce the overall injury rate among youth athletes in team sport by approximately 40% [8, 9, 10]. However, injury prevention programs are rarely implemented in real‐world practice, despite their benefits [11, 12, 13, 14]. Research demonstrates that the implementation can be negatively influenced by the deficiencies of coaches', athletes', and parents' knowledge and behavior [13, 15]. Likewise, interpersonal factors such as social support or sociocultural factors like norms and institutional forces have been proven to influence injury management (i.e., rehabilitation and return to sport) [16].

Throughout the last decade, reviews demonstrated how psychological, contextual, and social factors influence injury management at both youth and senior levels [17, 18]. In these, several factors have been identified (e.g., social support and the sport culture). In line with these reviews, a recent editorial in British Journal of Sports Medicine by Veith et al. [19] pointed to the importance of looking at the collaboration of all involved stakeholders in a sporting environment, and how these relations influence the health and performance of the athletes. Unfortunately, Truong et al. [17] argue that there is a tendency in the literature to primarily focus on factors related to the individual, and to investigate psychological and social factors together (i.e., psychosocial factors). Limiting focus to the individual level is at odds with a nuanced understanding of the fields.

Traditionally, injuries have been investigated at the individual component level [20] focussed on areas such as: the effectiveness of injury prevention programs [21], training loads' effect on injuries [22], biomechanic risk factors (e.g., of ACL injuries [23]), and psychological risk factors (e.g., stress [16]). Newer trends have suggested that injuries are the outcome of complex interplays between the individual, the physical surroundings, and the social relations [20, 24, 25], which is a reason to shift focus from the injured individual towards the context within which the individual is situated when injured and working to return to play [26]. To understand how the environment as a whole influences musculoskeletal injury prevention and management, researchers call for new approaches in injury research [26, 27, 28]. An “ecological (holistic‐developmental) approach” [29] (p. 136) has been suggested to facilitate an understanding of injury risk as embedded in the wider environment.

The holistic ecological approach (HEA) [30] is a framework rooted in ecological psychology [31], systems theory [32], and cultural psychology [33]. Ecological refers to a focus on the athletes' environment that influences their development, whereas holistic refers to viewing the environment as a complex and dynamic whole consisting of interrelated settings, levels, and domains [30]. HEA has been used to emphasize a contextually situated understanding of talent development in research. This approach emphasizes that athletic development is the result of complex interactions between the individual, their environment, and the sociocultural context over time. In HEA, an environment is defined as *a dynamic system consisting of a micro system, that is the athlete's immediate surroundings and the interrelations between these surroundings, and a macro system, which is the larger context in which these surroundings are embedded* [30]. Several features of the environment have been found to influence athletic development in both positive and negative ways [34].

Based on HEA, in this scoping review, we shift the object of analysis from individual level towards the wider environment, with the objective to identify positive and negative environmental factors associated with injury prevention and management in youth sport. The scope of this review includes all activities related to prevention and management of injuries (e.g., injury prevention programs, rehabilitation, and return to sport) and encompasses both acute sudden onset and repetitive gradual onset injuries [35].

## 1 Methods

### 1.1 Study Design and Registration

A scoping review was deemed appropriate to examine the research objective of this study. Scoping reviews are suitable to integrate different methods, to explore the existing literature, map the evidence and identify research gaps within the research area [36, 37]. This review was conducted according to the guidelines of The Joanna Briggs Institute [38] and reported according to the PRISMA‐ScR checklist [39]. The review was pre‐registered in Open Science Framework (https://doi.org/10.17605/OSF.IO/86SRT).

### 1.2 Search Strategy

A preliminary search was done in Scopus in August 2024 to develop the search terms and Boolean Operators. Words in titles, abstracts, and keywords from relevant studies were used to further specify the search string. The search string was reviewed by experienced researchers within sports psychology and sports medicine and discussed with a librarian. The final search string can be found in Appendix. The search string was adjusted to the five different electronic databases, which were deemed relevant: Embase, Medline, Scopus, PsycInfo, and SPORTDiscus. No start date of the timespan search was made to ensure a comprehensive search. The search was done on the 1st of November 2024. An updated search was done on the 31st of October 2025.

### 1.3 Selection Process

**FIGURE 1:**
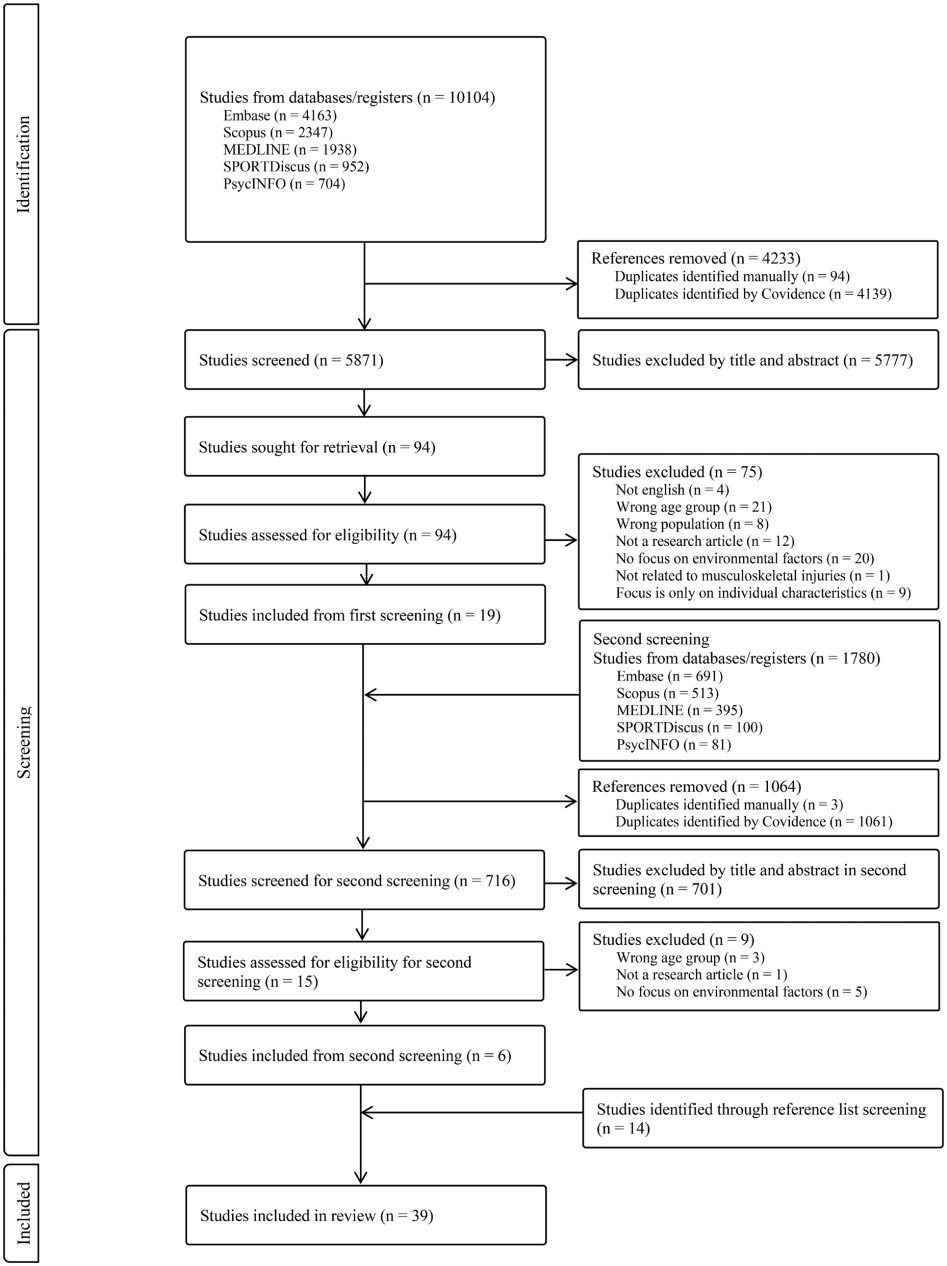
Selection process presented in a PRISMA‐ScR flow chart.

All identified studies were exported into the software Covidence [40]. The software enabled an automated removal of duplicates. The remaining hits were screened by title and abstract independently by NV and a student assistant based on the listed inclusion and exclusion criteria (see Table 1). As the objective of this scoping review was to map existing knowledge rather than quantifying, evidence synthesizes (i.e., reviews) were included, despite the risk of duplication of data [41]. All conflicts were discussed between NV, LS, and the student assistant. If studies included groups with participants beyond the age of 18 years, inclusion was based on mean age or discussed between NV, LS, and the student assistant. At full‐text screening, reasons for exclusions were noted (see Figure 1). The full‐text screening was done by NV and the student assistant, and conflicts were discussed with LS. Studies were included if they identified, described, or assessed an environmental factor at any stage of the injury process (i.e., prevention, acute, rehabilitation, and return to sport). Following this, the literature list of all included studies was checked.

**TABLE 1:** Study inclusion and exclusion criteria.

Inclusion criteria	Exclusion criteria
Publications in peer‐reviewed journals	Books, book chapters, gray literature
Quantitative, qualitative, mixed methods, reviews	Theoretical papers, positions statements
Relevance to the research question: Studies that can inform our understanding of the role of the youth sport environment in injury prevention and management All organized youth sport settings (e.g., talent development setup and recreational sport) Investigation of environmental factors within the sporting and/or non‐sporting domain Focus on musculoskeletal injuries Focus on youth sport (athletes aged 7–18)	Relevance to the research question — not incl.: Focus on individual characteristics of people in the environment (e.g., coach competences and treatments providers level of education)

### 1.4 Data Extraction

Data extracted from each study included: authors, year of publication, country of origin, aim, design, theoretical perspective, participants (sample size, age, gender, sport, and level), possible other participant groups, methods, data analysis, and key findings. In some articles, findings were related to both environmental factors and other factors (e.g., individual psychological characteristics). In these cases, only findings related to environmental factors were extracted. Data extraction was performed by NV using a premade extraction form. Specific for the column Framework, we identified what the authors stated as their theoretical framework (i.e., guiding data collection or analysis) based on the full‐text readings.

### 1.5 Data Analysis

Data were analyzed with a qualitative content analysis, as suggested for scoping reviews [41, 42]. Inspired by the main categories outlined in HEA [30], and through an iterative process, we categorized the findings into *Preconditions, Processes, Structure, and Culture*. This was an adjustment made after the protocol, as the initial model divided by systems (i.e., micro‐, meso‐, exo‐, and macro system) was insufficient in structuring the identified factors. *Preconditions* describes human‐, material‐, and financial resources, that were identified in the included studies. *Processes* refer to the everyday activities in the athletes' environments in both the sporting and non‐sporting domains. *Structure* comprises the components, roles, and relationships in the environment, and their organization at both micro and macro levels. *Culture* is the shared norms and values that shape and regulate the beliefs and behavior of people in the environment. Sub‐categories were also based on HEA. For example, under *Structure*, *Organizational level* consisted of factors in micro‐ and meso system (e.g., club and school), while *Sports system* consisted of factors in macro system. In *Processes*, all factors identified happened at *Team level* in the micro system, but because a substantial part of the studies focused on support, this was added as a further subcategory. The categorization of the findings was discussed with the research team until agreement was reached on the presented interpretation.

## 2 Results

### 2.1 Selection and Inclusion of Studies

The systematic search in the five databases generated a total of 10.104 hits. The software removed 4.139 duplicates automatically, and 94 duplicates were removed manually. After title and abstract screening, 94 hits were included for full‐text screening. In total, 19 articles were included, and an additional 12 studies were included based on literature list checking. After the updated search, 719 new studies were identified, from which six were included. After checking the literature lists of these six studies, two additional studies were included. In total, 39 studies were deemed suitable according to the inclusion criteria.

### 2.2 Study Characteristics

**FIGURE 2:**
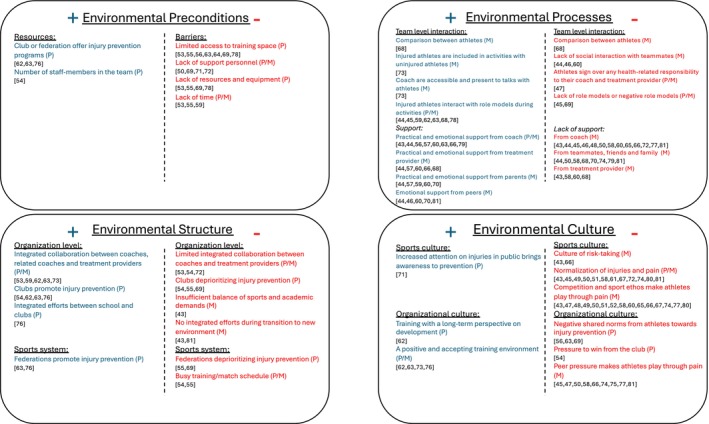
A mapping of the identified environmental factors related to injury prevention and management in youth sport. (P) = Studies related to injury prevention, (M) = Studies related to injury management. Blue color and “+” denotes a positive environmental factor. Red color and “–” denotes a negative environmental factor.

An overview of the 39 included studies is provided in Tables 2 and 3. In total, five studies were quantitative, 23 were qualitative, six were mixed methods designs, and five reviews were based on either only qualitative methodology (1) or both qualitative and quantitative methodology (4). The included studies originated only from Western countries: USA (12), Sweden (12), Norway (4), Canada (3), Australia (2), Germany (2), the United Kingdom (2), Ireland (1), and Switzerland (1). Of the included studies, 17 focused on athletes' and parents' perceptions of how factors of the environments influenced the injury process, and 10 focused on facilitators and barriers for injury prevention programs. Eleven studies focused on environmental factors that influenced injury prevention and management from other perspectives (e.g., ethnographies of the culture in youth sport or coaches' and treatment providers' perceptions). Fifteen studies were based on theoretical perspectives (e.g., Self‐determination theory or Theory of planned behavior; see Tables 2 and 3). Inspired by Hauser et al. [34], Figure 2 provides an overview of the identified environmental factors that influence injury prevention and management in youth sport.

**TABLE 2:** A summary of the identified review‐type articles. Wording of the data extracted is reformulated by the authors to create uniformity in data.

Author and year	Aim/focus of the review	Amount and characteristics of studies included	Contributions	Original studies also included in current review
Bjørndal et al. (2024) [43]	To identify and summarize how interpersonal and contextual dynamics influence the risk of youth sports injuries.	15 studies that focused on youth sports aged 10–19 years and injuries. All studies were qualitative.	Interpersonal dynamics: coach‐athletes relationship (e.g., getting support or being pressured), physicians‐athlete relationship, (in)appropriate balance between sports and academic requirements and leisure time, and the (insufficient) coordination across different contexts. Contextual dynamics: normalization of injuries and pain, sport culture of risk‐taking behavior, transition to new environment, socialization process of competitive sports, and the sport's ethos influencing athletes' behavior.	[44, 45, 46, 47, 48, 49]
Hausken‐Sutter et al. (2021) [50]	To examine multidisciplinary literature on youth sport injury etiology.	Studies that focused on youth sport etiology with athletes aged 10–19 years. Quantitative, qualitative studies and reviews.	The following elements were presented to potentially influence injury etiology: team climate (e.g., lack of support from coach and teammates), sociocultural values (e.g., the importance of sporting success), pressure to play through injury and pain, acceptance of training/competing with pain, silence around pain and injury, lack of medical support, coach‐athlete relationship.	[48, 49, 51, 52]
Hawkinson et al. (2022) [53]	To explore and summarize youth sport coaches' knowledge, beliefs, and contextual perceptions of injury prevention training programs.	19 studies that focused on youth sport coaches working with athletes under 18 years old. Quantitative and qualitative studies.	Coaches cited lack of time, space, athlete interest, support and resources as barriers to implementation of injury prevention.	[54, 55, 56]
Hoang et al. (2025) [57]	To discuss sport‐related injuries in children and adolescents regarding psychosocial factors, recovery, and prevention.	40 studies included with athletes under 18 years old. Quantitative, qualitative studies and reviews.	Healthcare practitioners provide appropriate support. Coaches and parents play a key role in reducing the risk of injury.	None
Podlog et al. (2024) [58]	To review the impact of injuries on parents and review how injury can impact parent transactions with relevant stakeholders.	49 studies included. Quantitative, qualitative studies and reviews.	Parents normalize demands, challenges and even abusive behaviors experienced by their adolescents. Parents experienced a lack of communication with coaches and healthcare providers during injury processes. Parents silenced their concerns related to adolescent's injuries. Athletes reported that they had to suppress injured‐related emotions within their team climate. Parents pressure adolescents to expedite their return to play. Some parents believe that it is needed to play with pain and injuries.	[59, 60, 61]

**TABLE 3:** A summary of the identified original articles. Wording of the data extracted is reformulated by the authors to create uniformity in data.

Author and year	Major foci	Theoretical framework	Participants and contexts	Methodology	Major findings
Ageberg et al. (2019) [62]	Identifying facilitators to support implementation of IPP.	The Translating Research into Injury Prevention Practice (TRIPP) and the seven steps for Implementing Injury Preventive Training	Brainstorming: 57 athletes, 26 coaches, 75 caregivers, 11 club administrators, 10 district/national administrators, 17 unknowns (total: 196). Sorting and rating: 17 players, 11 coaches, 12 caregivers, 5 club administrators, 5 district/national administrators (total: 50). Youth community handball in Sweden.	Mixed method. Concept mapping.	Important that injury prevention training becomes an integral element and is mandatory from the club (e.g., included in syllabus of education). To have a long‐term perspective on training, for life and the individual. Teach youths to train for the long term and with an all‐round approach including recovery. Create a training environment which is positive, accepting and fosters development. Coaches are to collaborate with physical therapists. Demonstrate good examples of rehab if injury occurs and ensure that the players receive support in the process.
Åkerlund et al. (2024) [63]	Athletes' and coaches' use of IPP and barriers to program use.	Not specified	42 athletes (24 females), 12 coaches (5 females). Youth floorball, Sweden.	Qualitative. Focus group interviews.	Negative attitudes from players affect others' commitment to IPP. Limited access to court limits preventive training. Coaches feel alone in prioritizing preventive training with limited support from other coaches. Coaches offering support and feedback supports implementation of IPP. Teammates supporting each other. Coaches sharing knowledge with other coaches. Support from club and federation to do IPP. Role models to create a positive culture around preventive training.
Åkerlund et al. (2023) [64]	Facilitators and barriers to use of IPP, and factors associated with continuous use.	The Health Action Process Approach	246 athletes (93 females), 35 coaches (12 for females' teams). Youth floorball, Sweden.	Quantitative. Cross‐sectional survey.	Lack of space to do exercises is a barrier for implementation of injury prevention training.
Barker‐Ruchti (2008) [65]	Gymnasts' daily training experiences.	Foucault's concept of modern social control	6 athletes (all females) and their two coaches (some athletes under 18). Elite Artistic gymnastic, Australia.	Qualitative. Ethnography.	Sport ethos makes athletes play through pain and injuries. Coaches' leadership style makes athlete hide pain and play through injuries.
Barette & Harman (2020) [66]	Athletes', coaches' and rehabilitation specialists' view of playing through pain, and roles and decision making in the team.	Not specified	4 athletes, 5 coaches, 3 rehabilitation specialists. Subelite, gymnastics, rowing and speed skating, Canada.	Qualitative. Ethnography. Observations and interviews.	Peer pressure makes athletes play through pain and makes athletes feel bad when they withdraw from practice. The importance of competitions makes athletes play through pain. Culture of risk‐taking was a source of pressure. People celebrate those who are though. Relationship with coach and rehab specialist can facilitate the rehab process. Athletes describe that they are taught to hide their pain.
Cavallerio et al. (2016) [49]	Sport culture in rhythmic gymnastics and how it impacts overuse injuries.	Not specified	16 athletes, 3 coaches, 1 physiotherapist, 22 parents, and 1 club president. All from the same club. Elite rhythmic gymnastics, Italy.	Qualitative. Ethnography. Observations, interviews, focus group interviews.	Socio‐cultural values leading athletes to act a “mentally tough” attitude and behavior, that meant that they accepted pain as part of the sport and continuing to train and compete despite pain.
Cavallerio et al. (2022) [61]	Experiences of parents of gymnasts suffering from overuse injuries.	Not specified	16 athletes, 3 coaches, 1 physiotherapist, 22 parents, 1 manager Elite rhythmic gymnastics, Italy.	Qualitative. Ethnography. Observations, interviews, focus group interviews.	Parents strive to adapt their support to their daughters in rhythmic gymnastics, who is suffering an overuse injury while being part of a culture that normalizes pain and injuries.
Curry et al. (1993) [67]	Acceptance of pain and injuries in amateur wrestling.	Role‐identity approach	1 athlete. Elite amateur wrestling, USA.	Qualitative. Interview.	Injuries are normalized as a part of sport. Sport ethos makes athlete practice through pain and injury.
DiSanti et al. (2018) [68]	ACLR‐injured adolescents' perception of the rehabilitation and RTS process.	Not specified	10 athletes (aged 15–18 years; 7 females). With a ACLR no more than 12 months prior to interview. Basketball, soccer, football, volleyball, skiing, ice hockey, and lacrosse. USA.	Qualitative. Interviews.	Barriers for returning: Interpersonal comparison with other people rehabbing. Positive recovery factors: Trusting relationship with physical therapist or athletic trainer. Comparison with other people rehabbing, who were longer in the process. Role models who were back to normal function. Negative recovery factors: Social interactions where people treated their RTS with hesitation and caution. Lack of attention and guidance from physical therapist or surgeon.
Donaldson et al. (2019) [69]	Barriers to implementing an IPP.	Not specified	17 participants (9 females) involved in soccer (mean age = 42.7). 7 coaches, 6 federation members, 4 others. Youth soccer, Australia.	Mixed method. Concept mapping.	Lack of support personnel. Limited training space provided. Lack of resources and equipment. Limited information from the club. Lack of leadership from federation. Lack of support from federation. Lack of visible leadership from recognized role models in the sport on value of injury prevention. Club leadership does not prioritize player welfare. Club/team culture that undervalues physical/psychological preparation.
Golub & Steinfeldt (2025) [70]	Athletes' experiences with multiple injuries.	Biopsychosocial Model of Sport Injury Rehabilitation	8 athletes (all males) with multiple injuries aged 14–18 years (mean age = 15.6). American high school football, USA.	Qualitative. Interviews.	Support from parents. Support from their teammates. Lack of support from coaches and teammates. Seeking up support persons during rehabilitation.
Hallquist et al. (2016) [59]	Coaches', physiotherapists', and parents' perception of adolescents' psychosocial support needs during injuries.	Not specified	Coaches, parents, and physiotherapists of adolescents aged 12–16 years, who had experience of at least one severe sport injury. Handball, football, team gymnastics, artistic gymnastics, and ice hockey, Sweden.	Qualitative. Interviews.	Coaches could not provide the care needed due to lack of time. A mentor for the athlete with similar injury is beneficial. Family's support is important. Collaboration between coach and physiotherapists supports rehabilitation.
Ingram et al. (2025) [71]	Coaches' view of youth sport culture and their role as a coach.	Not specified	30 coaches (28 males; mean age = 46.2; mean years coached = 12.1). Baseball, basketball, football, hockey, lacrosse, soccer, softball, and tennis, USA.	Qualitative. Focus group interviews.	Research efforts and media attention on injuries help to bring awareness to and implement injury prevention. Lack of support personnel is a barrier for athletes' health. Support from organizing bodies is a facilitator for athletes' health.
Jacobsson et al. (2018) [52]	Factors related to injuries and other health problems in track and field.	Not specified	79 participants (45 females) working within youth sport. A mix of athletes, retired athletes, coaches of youth sport, coaches of senior sport, parents, medical persons, board members and others. Track and field, Sweden.	Qualitative. Focus group and individual interviews.	The interpersonal relation and coordination between coaches, parents, and medical practitioners directly affect the athlete. “Short sighted”‐ youth athletics community. Training lacks a long‐term developmental perspective. The championship paradigm is introduced too early. Adult elite like training and competitions culture for kids.
Jacobsson et al. (2023) [72]	The knowledge and understanding of injuries among sport high school students and their needs managing health problems.	Not specified	119 athletes (61 female) aged 16–19. All part of a Swedish sports high school. Track and field, Sweden.	Qualitative. Focus group interviews.	A culture of acceptance that injuries will occur within the sport. Inadequate communication with/between coaches about injuries make athletes experience stress or frustration. Lack of access to support persons.
Kempe et al. (2023) [73]	Sport high school coaches' perception and experience of injury prevention.	Not specified	10 coaches (2 females) at Swedish sports high schools aged 29–59 years. Track and field, basketball, cross‐country skiing, football, handball, orienteering, swimming, triathlon, and volleyball, Sweden.	Qualitative. Interviews.	Include injured athletes with the uninjured athletes. Having an open and safe environment where athletes can share their thoughts and feelings with the coach. Collaboration between coach and medical personnel is important for implementing injury prevention. Coach being accessible and present to talks with athletes.
Kerr et al. (2023) [74]	The sport culture in middle school sports and communication strategies.	Not specified	19 athletes aged 12–14 years, 20 parents, 18 staff. Different sports (not specified what sports the athletes participated in), Middle School, USA.	Qualitative. Interviews.	Competitiveness makes athletes not reporting injuries, because they don't want to sit out games. Athletes view injuries as a part of sport. Athletes feeling pressured to continue playing despite injuries. Parents expect athletes to play through injury.
Kristensen et al. (2023) [75]	Social pressure and intention to play injured.	Theory of planned behavior	186 athletes (23 females) aged 16–20. Ice hockey, Norway.	Quantitative. Survey.	Perceived social pressure predicts intension to play injured.
Kuhlin et al. (2020) [48]	A figure skater's narrative of self, coach‐athlete relationship, personal development, and career.	A socio‐narratology approach	A retired athlete. Figure skating, Sweden.	Qualitative. Self‐narrative.	Pressure from coach makes athlete train despite being injured or sick.
Lindblom et al. (2018) [55]	Factors influencing coaches' adoption of IPP.	The Health Belief Model and an ecological perspective	20 football coaches for athletes aged 10 to senior level in Sweden. Mean age 44 (range: 23–52) Female youth football, Sweden.	Qualitative. Interviews.	Limited support from club or federation negatively influences the use of IPP. Limited financial resources, time, access to indoor and outdoor venues and coach availability negatively influence the use of IPP. Tough training schedule contributes to injury occurrence.
Luteberget et al. (2025) [76]	Identify facilitators for implementing injury prevention.	Not specified	Stakeholders (Coaches, Administrators, Health Staff, Players) from U14‐U18. Brainstorming: 224 stakeholders. Sorting: 47 stakeholders. Rating importance: 57 stakeholders. Rating feasibility: 49 stakeholders. Handball, Denmark.	Mixed method. Concept‐mapping.	An accepting training culture in which it is legitimate to speak up about pain or injuries. A strategy from the federation supporting injury prevention. Knowledge about injury prevention is provided to clubs. Clubs' policy about injury prevention is implemented at all age groups. Injury prevention is made compulsory. Clubs prioritize a health sector. Cooperation between schools and clubs. Federation creates requirements for clubs to ensure injury prevention is prioritized.
Malcom (2006) [51]	The culture in female softball in relation to injuries and pain.	Socialization of the Sport Ethic	Players and coaches (number not specified) Recreational, female, youth softball, USA.	Qualitative. Ethnography. Observations.	Players' reaction to pain and injuries are influenced by the behavior of the coach and the culture of the sport. Players adopt the sport ethics about “shaking it off” or “toughing it out”.
Mayer et al. (2018) [77]	Adolescent athletes' willingness to compete hurt	Willingness to compete hurt	1138 (500 females) athletes aged 14–18. Participated in one of four national squad levels. Olympic sports, Germany	Quantitative. Survey.	Peer‐pressure towards playing hurt. Fear of being called a weakling can make athletes play hurt. Coaches' leadership style can lead to athletes playing hurt.
Moesch et al. (2022) [78]	Coaches' and athletes' experience with IPP.	Not specified	12 athletes aged 14–16 (5 females), 5 coaches (1 female). From same two clubs. Youth handball, Sweden.	Qualitative. Focus group interviews.	Role models is a motivating factor to do IPP. Limited space and materials can negatively influence use of IPP.
Munoz‐Plaza et al. (2021) [56]	High school coaches' and players' practice, knowledge, and perspectives about warm‐ups and injuries.	Not specified	12 coaches (3 female) for single interviews and 30 of the coaches' players (aged 14–18; 19 females) for focus group interviews. Varsity basketball, high school, USA.	Qualitative. Single and focus group interviews.	Access to space and players not taking warm‐up seriously are barriers for warm‐up engagement. Coaches' engagement is a facilitator for implementation of warm‐up activities.
O'Brien et al. (2016) [54]	Coaches', fitness coaches' and physiotherapists' perception towards IPP and maintenance of these.	Reach Effectiveness Adoption Implementation Maintenance and Health Belief Model	9 coaches, 4 fitness coaches and 5 physiotherapists. Junior elite male soccer, Europe.	Quantitative. Cross sectional survey.	Barriers to IPP delivery: Lack of communication and teamwork between team staff. Lack of structure and support from the club. Pressure to win from the club. Heavy game schedule from the governing bodies. Facilitators for IPP delivery: Club structure and support. Incorporated into club policy. Regular team meetings focusing on prevention and long‐term planning. Available staff members.
Podlog et al. (2012) [60]	Parents' perception of adolescents' rehabilitation and RTS experience and their own role in the rehabilitation process.	Self‐determination theory	10 parents (aged 39–51 years; 7 females) of athletes aged 12–17 at state team or institute of sport squad level and that have had a minimum one‐month absence from sport due to injury. Basketball, netball, soccer, rowing, and track and field, Australia.	Qualitative. Longitudinal interviews.	Missed social interaction with teammates negatively influence athletes' well‐being and self‐concept. Taking on a different team role or to support teammates can help the rehabilitation process. The macho sporting ethos, where athletes must hide or disregard injury pain challenge the rehabilitation process. Coaches putting pressure on athletes to return to sport. Not getting support from coaches or physio influence rehabilitation. Social support from parents, teammates and coaches help the rehabilitation process.
Podlog et al. (2013) [44]	Adolescent athletes' perspectives of their rehabilitation and return to sport experiences.	Self‐determination theory	11 athletes aged 12–17 (8 females). From academy squad or a state/national team. With musculoskeletal injury requiring a minimum 1‐month absence. Basketball, netball, soccer, rowing, and track and field, Australia.	Qualitative. Interviews.	Isolation from the rest of the team negatively affected the rehabilitation process. Social support from parents, teammates, peers, coaches, sport medicine specialists, and role models was important. Support from role models provided sense of comfort and reassurance. Pressure from friends and parents to make an expedited return to sport negatively influenced the rehabilitation process. Coaches encouraging athletes to take time to recover supported the rehabilitation process.
Solstad et al. (2025) [79]	Investigate prevalence of injuries and the relationship between prevalence and risk factors.	Complex System Approach	568 athletes (males: *n* = 386) players from U14‐U18 teams. Grassroot soccer, Norway.	Quantitative. Cross‐sectional survey.	Lower levels of perceived peer autonomy support showed higher prevalence of injuries. Higher levels of autonomy support from the coach showed a lower prevalence of injuries.
Thiel et al. (2015) [47]	Psychological and social factors influencing injuries.	The Biopsychosocial perspective on health	Survey: 1138 athletes. Case studies: artistic gymnastics, biathlon, handball, and wrestling. Interviews with 50 youth elite athletes, coaches, and physicians. Olympic sports, Germany.	Mixed methods. Survey. Case studies: interviews and observations.	Socialization process where athletes “incorporate” the sports specific culture of risk; they seek the ability to ignore pain and avoid expressing pain. Peer pressure made athletes joke about pain. Athletes transferred the health‐related responsibility to their coach and physiotherapists.
Von Rosen et al. (2018) [46]	Adolescent athletes' perceptions and experience of being injured.	Not specified	Survey: 340 athletes (155 females) from sports high schools (age 15–19). From 16 different sports. Focus group: 20 athletes (12 females) from 10 different sports. Sweden	Mixed methods. Survey and focus group interviews.	Being excluded from the social sport context was perceived as a negative experience when injured. Limited support from coaches was a negative experience during rehabilitation.
Wall et al. (2023) [45]	Adolescent athletes' experience with sport‐related low back pain.	Not specified	9 athletes (5 female) aged 14–19. Rowing, cycling, hurling, Gaelic football, rugby, American football, basketball, lacrosse, and competitive cheer, Ireland and USA.	Mixed method. Survey and interviews.	The culture of normalizing low back pain in sport negates safeguarding efforts aimed at protecting athletes against injury and pain. Athletes hiding their pain for fear of how they would be perceived by others. Role models can both be positive and negative. Coaches asking players to continue in practice despite pain, makes the athlete feel, that the team's success is priorities over them as individuals.
Yeldon & Pitter (2017) [80]	Young athletes' perception of acute and chronic pain.	Not specified	12 athletes (All boys) aged 9–13. Ice hockey, Canada.	Qualitative. Focus group interviews.	Pain and injuries are seen as normal in sport. Sporting ethos and competition make athletes hide their pain and injuries.
Zwolski et al. (2024) [81]	Athletes' perceived facilitators and barriers for physical activity after ACLR.	Not specified	10 athletes (aged 17–28; age at time of injury 12–18). Basketball, volleyball, track and field, lacrosse, football, soccer, swimming, USA.	Qualitative. Interviews.	Peer support facilitated the rehabilitation process. No/limited support lessened the desire to return to sport. Transitions in other parts of life (school, work) led to decreased physical activity.

Abbreviations: IPP, Injury Prevention Program; RTS, Return to Sport.

### 2.3 Preconditions

Preconditions are divided into two subcategories: *Resources* (i.e., that positively influence the prevention and management of injuries) and *Barriers* (i.e., that negatively influence).

#### 2.3.1 Resources

Only four studies identified resources as positive factors. Clubs and federations that offered sport‐specific injury prevention programs to coaches were found to influence the implementation of these [62, 63, 76]. These programs supported coaches' knowledge about injury prevention programs and made it easier for them to implement. The total number of staff members working with the athletes was also found to positively influence the use of injury prevention programs [54].

#### 2.3.2 Barriers

Lack of human‐, material‐, or financial resources has been identified as a negative factor in the literature. Lack of time influenced coaches' implementation of injury prevention programs [53, 55] and limited their support to injured athletes [59]. Limited time and access to sufficient training facilities were identified as barriers for coaches' use of injury prevention programs [53, 55, 56, 64, 69, 76, 78]. The lack of (the right quality) material and financial resources also negatively influenced the implementation of injury prevention programs [53, 55, 69, 78], whereas the lack of support personnel both influenced the implementation of prevention programs [69], and influenced athletes' rehabilitation process negatively due to limited support [50, 71, 72]. Overall, lack of resources has received more attention in the literature than resources as positive factors.

### 2.4 Processes

Processes are divided into the two subcategories *Team Level Interaction* (i.e., activities between the athlete and other participants related to the team) and *Support* (i.e., an essential part of the injury process).

#### 2.4.1 Team Level Interaction

Comparison with peers has been identified as both a positive and a negative factor for the rehabilitation process. When positive, the injured athlete compared oneself with teammates facing similar challenges, fostering learning through shared experiences [68]. On the other hand, comparison made athletes feel frustrated if they compared themselves with athletes who were longer in the injury process [68]. Likewise, having role models in the daily activities was important, as they showed how to do appropriate injury prevention or how to handle rehabilitation [44, 45, 59, 62, 63, 68, 78]. On the contrary, the lack of role models, or even negative role models that did not prioritize injury prevention, constrained the athletes' rehabilitation process or their perception of injury prevention [45, 69]. Including injured athletes in activities with uninjured athletes was found to be positive for the athlete's rehabilitation [73], as rehabilitation often isolated the athlete from the rest of the team, leading injured athletes to miss social interaction with their teammates, making them feel left out and alone [44, 46, 60]. Thus, an unstructured rehabilitation process can influence the injured athlete negatively. Coaches who were accessible and present to have talks with injured athletes facilitated adjustment of the training load and support to athletes [73]. A risk was identified during the rehabilitation process that athletes would transfer the health‐related responsibility to their coach and treatment provider [47], which fostered dependency on external support and reduced their own agency in health‐related activities.

#### 2.4.2 Support

Support, or the absence of it, was identified to influence injury prevention and management. While 18 studies identified support within the sporting domain, only eight studies identified support within the non‐sporting domain. Practical (e.g., getting treatment and the right exercises) and emotional support (e.g., getting attention) from the coach [43, 44, 56, 57, 60, 63, 66, 79] and treatment providers [44, 57, 60, 66, 68] have been identified to be a positive factor for athletes during prevention and management. Likewise, getting practical (e.g., rides to treatment providers) and emotional support from parents [44, 57, 59, 60, 70] or peers [44, 46, 60, 70, 81] also positively influenced management. Compared to the presence of support, lack of support has been identified more often in the literature. Lack of support from the coach [43, 44, 45, 46, 48, 50, 58, 60, 65, 66, 72, 77, 81], treatment provider [43, 58, 60, 68], teammates, friends, and family [44, 50, 58, 68, 70, 74, 79, 81] influenced management negatively. To be pressured by coaches, treatment providers, or parents to play despite being injured or in pain, or to return to sport before being fully ready, was described as a lack of support. Overall, the studies demonstrated that receiving support in both the sporting and non‐sporting domain was crucial for athletes' rehabilitation. If an athlete did not receive appropriate support, there was a risk that they would not return to the sport after the injury [81].

### 2.5 Structure

This category includes planned and structured roles, relationships, and initiatives. It is divided into the subcategories of *Organizational Level* (i.e., club‐level) and *Sport System* (i.e., federation and national sport policies).

#### 2.5.1 Organization Level

A structured setup where coaches collaborated with treatment providers or other coaches was positive for the implementation of injury prevention programs and during rehabilitation [53, 59, 62, 63, 73], whereas the lack of collaboration negatively influenced the implementation of injury prevention programs or prolonged rehabilitation [53, 54, 72]. Clubs prioritizing injury prevention by supporting coaches applying it in daily practices or by implementing it into club policy and syllabus of education was found to positively influence the use of injury prevention [54, 62, 63, 76]. On the contrary, limited support and focus from the club towards injuries was found to limit coaches' prioritization of injury prevention programs [54, 55, 69]. A study identified that cooperation between schools and clubs could facilitate injury prevention [76], while an imbalance and insufficient coordination between sport and academic requirement led athletes to making compromises about their injuries [43]. Transitioning to a new environment influenced athletes negatively, either by a sudden increase in training load (e.g., when moving to a higher‐level team) [43], or because transitions in the non‐sporting domains took focus away from rehabilitation (e.g., starting in a new school) [81].

#### 2.5.2 Sport System

Federations that support the use of injury prevention and have implemented injury prevention programs into coach education positively influenced the implementation of prevention in daily practices [63, 76]. The contrary was the case if there was limited support and focus from the federation regarding injury prevention [55, 69]. Finally, a busy calendar of training and competition was identified to increase the risk of injury occurrence [55] and to limit coaches' use of injury prevention [54].

### 2.6 Culture

This category refers to the culture of the youth sport environment. Culture includes two subcategories *Sports Culture* (i.e., cultural aspects that are common for sport in general) and *Organizational Culture* (i.e., team‐ and club culture).

#### 2.6.1 Sports Culture

Only one positive factor about general sporting culture was described across all the included studies. Increased attention on injuries in the media influenced coaches' use of injury prevention strategies in a positive manner [71]. In contrast, negative factors have been extensively investigated. A sports culture of risk‐taking was described as a disconnection between the athlete's capabilities and the demands imposed on them, leading athletes to feel pressured to do risky behavior [43, 66]. Across several different sports, a normalization of injuries and pain was described [43, 45, 49, 50, 51, 58, 61, 67, 72, 74, 80, 81]. The assumption that injuries and pain are an inevitable part of being an athlete was proved with examples of silenced athletes [45], or coaches framing injuries as an inherent and normal risk of sports [49]. The competitive nature of sport and the sport ethos that makes athletes play through pain were identified in numerous studies [43, 47, 48, 49, 50, 51, 52, 58, 60, 65, 66, 67, 74, 77, 80]. For example, studies described a sports culture that made athletes hide pain and injuries from coaches because of fear of missing competitions [49, 80], or hiding pain from their peers to avoid being perceived as weak [66, 77]. The norms and values embedded within the general sports culture permeated the organizational culture and thereby influenced the processes and decisions related to injury prevention and management.

#### 2.6.2 Organizational Culture

A study identified that a training culture with a long‐term developmental perspective can ease the implementation of injury prevention programs [62]. A positive and accepting training culture with athletes supporting each other during exercises and sharing their thoughts and feelings with support‐persons was positive for both prevention and management [62, 63, 73, 76]. Negative aspects of the organizational culture were identified in 13 of the 39 included studies. These studies found that negative shared norms from athletes towards injury prevention limited the quality of the execution of exercises and made it harder for coaches to implement [56, 63, 69]. Clubs prioritizing the importance of winning made coaches deprioritize the use of injury prevention programs [54]. Finally, several studies have described how peer pressure made athletes play through pain, which increased the risk of worsening their injury [45, 47, 50, 58, 66, 74, 75, 77, 81].

## 3 Discussion

The current scoping review identified 40 positive and negative environmental factors associated with injury prevention and management in youth sport including all activities (e.g., injury prevention programs, rehabilitation, and return to sport). We categorized the factors into preconditions (e.g., lack of resources and equipment), processes (e.g., role models), structure (e.g., integrated collaboration between coaches, related coaches and treatment providers), and culture (e.g., normalization of injuries and pain). Most studies were based on qualitative methodologies.

This scoping review is the first to apply HEA as a theoretical framework to map environmental features associated with injury prevention and management research. A few studies mentioned an ecological perspective in their sampling of participants [62], development of interview guide [55], or to put their own research in perspective [52], but no single study was identified that made the environment the central object of investigation. However, each study contributed fragmented insights into isolated environment variables. Collectively, they provide an initial sense of how the whole environment may or may not facilitate injury prevention, rehabilitation, and return to sport. Based on the current literature, however, we cannot say how these factors work together within a specific environment.

Our findings highlight that previous empirical research primarily focused on how environmental factors negatively influence injury prevention and management (e.g., [49, 52, 69]). For example, positive aspects of the sporting culture are under‐explored. A substantial part of the included studies found that the normalization of injuries and pain [43, 45, 49, 50, 51, 58, 61, 67, 72, 74, 80, 81], and competition and sport ethos [43, 47, 48, 49, 50, 51, 52, 58, 60, 65, 66, 67, 74, 77, 80] influenced prevention and management negatively. These cultural factors may exist as beliefs and assumptions that are no longer questioned by coaches and athletes and are therefore taken for granted. Cultural factors in the macro system can influence the behavior of athletes and coaches in the micro system indirectly without them being aware [82]. Talent development research based on HEA has previously described how a culture of long‐term development focus can facilitate successful athlete development [83], whereas a culture of short‐term result focus can influence the development negatively [84]. As only one positive aspect of the sporting culture was identified in the literature, we encourage researchers specifically to investigate aspects of sporting cultures that are positively related to injury prevention and management (e.g., being goal directed through rehabilitation).

Our scoping review included environmental factors associated with both the injury prevention and management processes, while previous reviews of psychosocial [18], and social and contextual factors [17] focused exclusively on the process of management of injuries (e.g., rehabilitation). In our scoping review, we identified a broad range of environmental factors. For example, our review highlights that the degree to which clubs and federations promote and prioritize injury prevention can influence the implementation of injury prevention in everyday practices both positively and negatively. Truong et al. [17] concluded that injury rehabilitation is influenced positively by social support, role models, supporting training groups, and negatively by the sporting culture and pressure from peers and support persons. Similarly, Forsdyke et al. [18] concluded that support is important for the athletes during rehabilitation, and that an environment where athletes do not feel safe to disclose their feelings was related to an impeded rehabilitation process. Our findings support but also nuance these ideas. For example, role modeling can also influence rehabilitation negatively, and we found factors related to the prevention of injuries not previously identified. Limiting our focus to youth sport (aged 7–18), whereas previous reviews had no specific age span, we contribute specificity for the youth sport context.

This scoping review suggests a comprehensive understanding of environmental factors influencing injury prevention and management. It supports the narrative review of Bjørndal et al. [43] but has a broader scope. Their review concluded that contextual dynamics shape athletes' and coaches' behaviors and practices and are deeply influenced by institutionalized competitive sports ethos, rules and norms. We confirm this conclusion and, with our broader scope, contribute a mapping of specific tangible environmental factors at micro and macro levels that facilitate and constrain injury prevention and management. While Bjørndal et al. exclusively reviewed qualitative research investigating young athletes' injury‐related experiences, our review included studies across methodologies and included the perspectives of all stakeholders in youth sport.

This review included studies from across different age groups (i.e., 7–18 years), purpose (e.g., leisure time and talent development), and phases of injuries (i.e., prevention and management). Despite this diversity, we identified similar environmental factors across contexts. As an example, normalization of injuries and pain was identified in both recreational sport [51] and elite sport/talent development [49]. While our objective was to map factors in youth sport in a broad sense, we encourage future studies to differentiate between specific age groups, purpose and injury phases.

Studies identified in this review spanned multiple disciplines (i.e., sports medicine, sport psychology, and sport sociology), with a diversity of methodological and theoretical perspectives. This review hereby showcased the multi‐disciplinarity of youth sport injury research, as previously highlighted by Hausken‐Sutter et al. [50]. While studies from all these disciplines spoke to different aspects of environmental influence, HEA acted as a suitable framework to provide a coherent mapping, and seems a promising guide for future empirical research.

### 3.1 Methodological Considerations

A strength of this scoping review was its foundation in a theoretical framework with a definition of an environment that supported both the search and analytical process of the review. Despite this methodological strength, this review is not without limitations. First, a notable proportion of the included studies (14 out of 39) were identified through the reference lists of already included studies rather than from the database search. While literature list screening is a valuable supplement, this notable proportion of studies suggests that the search string has not been sufficiently inclusive to capture the breadth of the literature. Although, as mentioned by Troung et al. [17], there is an inconsistency in terminology used to define social and contextual factors within the sport and exercise medicine field, making it challenging to include all terms in the search string. Second, the limited presence of studies using quantitative research (five quantitative and six mixed methods) may partly be explained by the inclusion criteria applied in this review. Third, review studies were included, increasing the risk of duplicate data [41]. To increase transparency, review studies were presented in a separate table (Table 2) with a column presenting original studies from reviews also included in the current scoping review. Fourth, the exclusion of non‐English articles might have influenced the characteristics of the included studies as all originated from Western countries.

## 4 Perspective

Our scoping review contributes to the body of research by providing an overview and structure of positive and negative environmental factors associated with injury prevention and management in youth sport. The factors were mapped out into preconditions, processes, structure and culture based on HEA. The factors related to either injury prevention, injury management, or both. While some factors influenced in a positive manner, research primarily focused on factors that influenced negatively. As the identified factors were examined in isolation, this provides us a fragmented insight into the influence on injury prevention and management. In this review, HEA served as a useful framework to map the environmental factors. We thus suggest HEA as a theoretical lens for future empirical studies. To derive an empirically based and cohesive understanding of how environments that handle the prevention and management of injuries are structured and cultivated, new designs and approaches are required. To this end, we suggest case studies of specific environments. Case studies are particularly suitable to explore the complexity of the phenomena in a bounded context [85]. Using multiple methods, a case study approach could provide an integrated perspective on preconditions, processes, structures and cultures associated with injury prevention and management within youth sport environments. The findings of this scoping review suggest that practitioners should attend to and improve athletes' broader environments, although further research is needed to guide this shift.

## Funding

This project was supported by a grant from Ministry of Culture Denmark (Grand ID: SUAKPKfor2W.2023‐010).

## Conflicts of Interest

The authors declare no conflicts of interest.

## Data Availability

Data sharing not applicable to this article as no new datasets were generated. The study is based on data from previously published articles.

## Search Strings

Scopus:

(((Environment* OR social OR context* OR ecological* OR milieu* OR culture* OR cultural OR barrier* OR “risk factor*” OR predictor* OR complexity OR biopsychosocial OR sociocultu* OR socioecological OR “system thinking”) W/6 ( sport* OR athletic* OR exercise OR “physical activit*” )) AND (injur* OR trauma* OR reinjur* OR accident* OR complaint* OR “health problem*” OR “physical problem*” OR pain*) AND (youth* OR adolescen* OR young* OR junior* OR teen* OR juvenile* OR “high school athlete*” OR “college* athlete*” OR “student athlete*”))

SPORTDiscus:

(((Environment* OR social OR context* OR ecological* OR milieu* OR culture* OR cultural OR barrier* OR “risk factor*” OR predictor* OR complexity OR biopsychosocial OR sociocultu* OR socioecological OR “system thinking”) N5 ( sport* OR athletic* OR exercise OR “physical activit*” )) AND (injur* OR trauma* OR reinjur* OR accident* OR complaint* OR “health problem*” OR “physical problem*” OR pain*) AND (youth* OR adolescen* OR young* OR junior* OR teen* OR juvenile* OR “high school athlete*” OR “college* athlete*” OR “student athlete*”))

Medline:

APA PsycInfo:

Embase:
